# Nanomolar Nitric Oxide Concentrations in Living Cells Measured by Means of Fluorescence Correlation Spectroscopy

**DOI:** 10.3390/molecules27031010

**Published:** 2022-02-02

**Authors:** Roksana Markiewicz, Jagoda Litowczenko, Jacek Gapiński, Anna Woźniak, Stefan Jurga, Adam Patkowski

**Affiliations:** 1 NanoBioMedical Centre, Adam Mickiewicz University, Wszechnicy Piastowskiej 3, 61-614 Poznan, Poland; anna.wozniak@up.poznan.pl (A.W.); stjurga@amu.edu.pl (S.J.); adam.patkowski@amu.edu.pl (A.P.); 2 Department of Molecular Biophysics, Faculty of Physics, Adam Mickiewicz University in Poznan, ul. Uniwersytetu Poznańskiego 3, 61-614 Poznan, Poland; jacek.gapinski@amu.edu.pl; 3 Department of Biochemistry and Biotechnology, Poznan University of Life Sciences, Dojazd 11, 60-632 Poznan, Poland

**Keywords:** nitric oxide, NO, nitric oxide determination, fluorescence correlation spectroscopy, FCS

## Abstract

Measurement of the nitric oxide (NO) concentration in living cells in the physiological nanomolar range is crucial in understanding NO biochemical functions, as well as in characterizing the efficiency and kinetics of NO delivery by NO-releasing drugs. Here, we show that fluorescence correlation spectroscopy (FCS) is perfectly suited for these purposes, due to its sensitivity, selectivity, and spatial resolution. Using the fluorescent indicators, diaminofluoresceins (DAFs), and FCS, we measured the NO concentrations in NO-producing living human primary endothelial cells, as well as NO delivery kinetics, by an external NO donor to the immortal human epithelial living cells. Due to the high spatial resolution of FCS, the NO concentration in different parts of the cells were also measured. The detection of nitric oxide by means of diaminofluoresceins is much more efficient and faster in living cells than in PBS solutions, even though the conversion to the fluorescent form is a multi-step reaction.

## 1. Introduction

Nitric oxide (^•^N = O, abbreviated NO), despite its structural simplicity, is an important free radical biomolecule with complex chemistry, which has a crucial role in several physiological and biological functions, e.g., the prevention of oxidative stress and cytokine activation, neurotransmission, and vascular homeostasis [1]. NO-based therapies exhibit great potential as antimicrobials and antivirals in the acceleration of wound healing and anticancer treatment [2,3,4,5]. More recently, the potential of inhaled NO was also explored in COVID-19 treatment, since it can stabilize oxidation stress, which is the major cause of comorbidity deaths [6,7,8]. The most recognizable area in which NO is known to have significant influence is the cardiovascular system, where it regulates platelet aggregation, adhesion and vascular tone, and blood pressure. Interestingly, NO also plays a crucial role in plant growth and defense and is beneficial in stimulating plant germination and greening, controlling iron homeostasis in plants, and improving plant tolerance to salinity, metal toxicity, temperature, and drought [9]. 

The primary source of NO in living cells is L-arginine, which is converted to L-citrulline under the influence of nitric oxide synthases (NOSs), with simultaneous NO release. Three NOSs are commonly found in mammalian organisms: neuronal NOS, endothelial NOS, and inducible NOS (nNOS, eNOS, and iNOS, respectively) [10]. The general mechanism of NO production in living systems has been reviewed lately, including reviews of the endogenous (enzymatic) and exogenous (preparative) sources of its formation [11]. The unique chemical and physical properties of NO—reactivity, a short half-life, and fast diffusion—limit the therapeutic efficiency of NO gas, but a variety of donor compounds have been widely investigated, not only to exploit the NO physiology but also to extend the release possibilities [12]. The last few decades have led to the development of various nitrocompounds with specific segments able to facilitate NO release in different times and in the presence or absence of a trigger (e.g., pH, susceptibility to oxygen, light, and/or temperature) [13]. The most commonly used NO donors are low-molecular-weight compounds, including organic nitrates, diazeniumdiolates, S-nitrosothiols, stimuli-responsive N-nitrosamines, metal nitrosyl complexes, and furoxans [13,14,15]. The current therapeutic application of NO donors is limited by pharmacodynamics, pharmacokinetics (i.e., the level of absorption and toxicity), and NO’s bimodal pro- and antitumorigenic role [13,16]. Great hope is placed in suitable NO carriers that will reach the right place, extend the life span of NO, and release the right amount of NO in a controlled manner. In particular, NO delivery by nanocarriers is considered very promising. Numerous systems have been reported as nanocarriers, especially liposome nanoparticles, polymeric nanoparticles, and bio-derived nanoparticles, as well as inorganic materials such as iron oxide, silica, metallic nanoparticles, and carbon nanotubes [12,15,17,18,19,20]. 

Although potential nitric oxide nanocarriers are designed to release NO at a specific place in a controlled manner over time, the NO concentration in a living system is difficult to control and remains a great challenge. The rapid decomposition of NO also makes its detection and quantification technically challenging, even though this same property allows for a broad range of analytical techniques to be employed. Two main groups of methods are usually considered: spectroscopic methods and electrochemical methods. The most common spectroscopic methods are (i) absorbance-based spectroscopy, (ii) fluorescence-based spectroscopy, (iii) chemiluminescence measurement, and (iv) electron paramagnetic resonance (EPR) spectroscopy, which has proven to be successful in NO detection in complex biological matrices [1,21,22,23,24,25]. Notably, a series of other approaches, e.g., gas chromatography, mass spectrometry, X-ray photoelectron spectroscopy, UV, infrared, and Raman spectroscopy, also proved to be useful; however, they never gained special attention [22]. 

A significant focus has been applied to the use of fluorescent indicators, diaminofluoresceins (DAFs). In contrast to techniques that require specific instrumentation and comprehensive knowledge, e.g., knowledge of chemiluminescence or EPR, DAFs are easy to use, inexpensive, and applicable with instruments that are widely available (fluorimeters, flow cytometers, and fluorescent microscopes) [26]. Fluorescence techniques have become the gold standard for NO-sensing because of their sensitivity and high spatiotemporal resolution when combined with microscopy [27]. Low concentrations (<1 nM) of NO dissolved in aqueous conditions of plant cell suspensions were found to be sufficient for precise quantification by chemiluminescence [25]. Still, the authors of that study could not measure DAF-2 fluorescence using a spectrofluorometer [25]. Specifically, in the case of biological imaging, it is important to note that the use of ozone or hydrogen peroxide is cytotoxic, whereas in EPR, a low spatial resolution is observed. Since the NO concentration is essential for its pro- or antitumorigenic effect (millimolar vs. pico- and nanomolar, respectively), and its rapid decomposition makes the detection process challenging, it is the responsibility of an appropriately sensitive and selective technique to verify the efficacy of NO delivery. Moreover, most detection techniques fail in obtaining precise NO measurements in a biological environment [13]. 

Due to the lack of selective and sensitive tools that can be used to measure intracellular concentrations of NO, the direct real-time measurement of NO in living cells is highly challenging, as discussed in many previous reports [21,28]. Recently, nanosensors based on poly(vinyl alcohol) with NO-responsive *o*-phenylenediamine-rhodamine for real-time direct imaging of endogenous NO in living cells were described [29]. However, that approach requires a long incubation period with nanosensor reagents, potentially prolonging the experimental procedure. 

This article addresses important questions concerning the possibility of measuring nanomolar NO concentration directly in living cells through fluorescence correlation spectroscopy (FCS). We used only a fluorescent dye, 4,5-diaminofluorescein diacetate (DAF-2DA), a well-known reagent for detecting NO in an aqueous solution. FCS was frequently used in previous studies of fluorophores in solution and has become a routine method for determining properties such as diffusion coefficients and viscosity, chemical rate constants, molecular concentrations, aggregation processes, fluorescence brightness, triplet state lifetimes, and other molecular parameters [30,31,32,33]. Much less frequent was the application of FCS in studies of living cells, even though it is an excellent analytical tool for studying the concentrations, propagation, interactions, and internal dynamics of various molecules at nanomolar concentrations [34,35,36]. FCS served as a valuable technique in systematic studies of nanoviscosity at different length scales [37,38,39], transport properties within cells with the use of fluorescent trackers [40], and interactions of different molecules [41]. For the current application, the most important features of the FCS technique are that it enables distinguishing between the mobile and immobile form of a dye, and that it determines the actual diffusivity and concentration of the free state. The measurement is limited to a sub-micron size of the confocal volume, which can be positioned at any chosen region of a living cell. In our case, FCS measurements were used immediately after adding the DAF2-DA fluorescent probe (10 nM to 1 μM), which significantly reduced the time of intracellular NO detection. To the best of our knowledge, this is the first time this technique was used to investigate nitric oxide release in living human primary endothelial cells and in the immortal human epithelial cell line.

## 2. Results and Discussion

### 2.1. Measurements of Nitric Oxide Concentration in DAF-2 + S-nitrosoglutathione(GSNO) /Glutathione(GSH) Mixture

To properly estimate the absolute concentrations of the DAF-2 in our sample, a calibration of the confocal volume was performed using Alexa Fluor 488 dye, taking its hydrodynamic radius as equal to 0.59 nm [42]. A value of *c_ref_* corresponding to *N* = 1 was calculated, using Equations (2) and (3), as *c_ref_* = 8 ± 1 nM. 

The reaction of the non-fluorescent form of the dye DAF-2 with nitric oxide leading to its fluorescent form DAF-2T was studied, using FCS in the DAF-2, S-nitrosoglutathione (GSNO) and GSH (glutathione) solution in a PBS (phosphate-buffered saline) buffer. The measurements of the DAF-2 solution in PBS indicated a residual fluorescence of this nominally non-fluorescent molecule. From the FCS measurements, the parameters CR = 4.28 kcts/s (the count rate), *N* = 0.78 (the mean number of fluorophores in the confocal volume), CPM = 5.62 (the counts per molecule), and *τ_D_* = 25 μs were obtained. These parameters defined the background for all further data. As this CPM value is too high for the non-fluorescent form of the dye, and the *N* value corresponds to the concentration of ~6 nM, it is probable that this signal resulted from the few DAF-2 molecules converted to the fluorescent form by the presence of nitric oxide amounts naturally occurring in water. At the equal concentration of all components of 50 nM, a slight increase in fluorescence intensity was observed, leading to the following parameters: CR = 6.65 kcts/s, *N* = 1.02, CPM = 6.84, and *τ_D_* = 27 μs. 

An increase in the GSNO concentration to 700 nM increased the fluorescence intensity, with the following parameters: CR = 7.09 kcts/s, *N* = 1.04, CPM = 7.35, and *τ_D_* = 24 μs. After 23 h, a further increase was observed: CR = 9.52 kcts/s, *N* = 1.55, CPM = 6.21, and *τ_D_* = 25 μs.

Thus, at a DAF-2 concentration of 50 nM and a GSNO concentration up to 700 nM, only a twofold increase in the fluorescence (above that of the background) was observed, mainly due to the increase in the concentration of fluorescing particles *N*. As *N* = 1 corresponds to the concentration of ~8 nM, only a very small part of the non-fluorescent dye molecules was converted to the fluorescent form after reaction with nitric oxide. 

**Figure 1:**
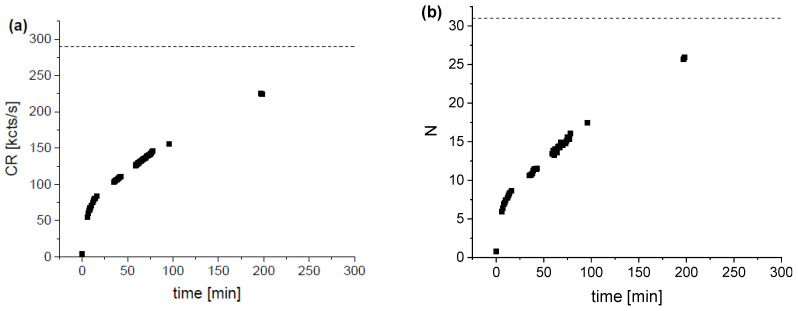
Plot of the time dependence of CR (**a**) and *N* (**b**) for the DAF-2 (250 nM) + GSNO (70 μM) solution in PBS. The dashed lines in (**a**) and (**b**) indicate the expected limits corresponding to *c_m_* = 250 nM.

To increase the efficiency of the reaction, the concentration of the DAF-2 was changed to 250 nM and GSNO to 70 μM and the kinetics of the reaction was measured. The results are shown in Figure 1.

As one can see, during 190 min, the fluorescence intensity (CR) substantially increased from the background value of ~5 kcts/s to ~225 kcts/s (Figure 1a), and at the same time, *N* increased from the background value of ~1 tFo to about 25 (Figure 1b). Since the CPM remains constant at about 9 kcts/s and, accordingly, the CR is a linear function of *N*, the increase in intensity is due to the increase in *N*, representing the concentration of fluorescent molecules. Using *c_ref_* = 8 nM, we found that approximately 80 % of the DAF-2 molecules were converted to the fluorescent form after 190 min. 

### 2.2. Measurements of Nitric Oxide Concentration in DAF-2 + SNP/GSH Solution in PBS

**Figure 2:**
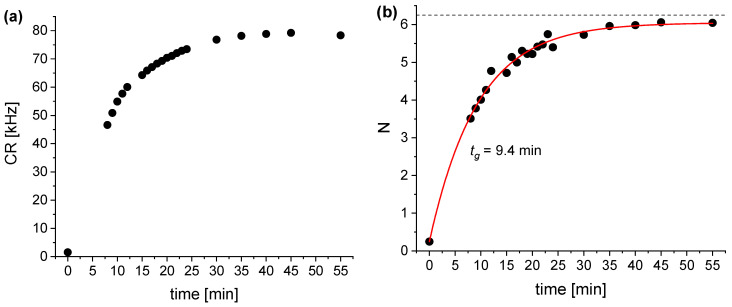
Plot of the time dependence of CR (**a**) and N (**b**) for the DAF-2 (50 nM) + SNP/GSH (14 μM) solution in PBS. The dashed line (**b**) represents the expected limit corresponding to *c_m_* = 50 nM. The solid line in (**b**) shows the inversed exponential decay fit with a characteristic time of 9.4 min.

The kinetics of the DAF-2 conversion to fluorescent form reaction, measured at a DAF-2 concentration of 50 nM and an SNP concentration of 14 μM, is shown in Figure 2. 

As shown in Figure 2a, the fluorescence intensity (CR) increased from the background value (DAF-2 in PBS) of 1.5 cts/s to 79 kcts/s within about 40 min. The initial part of the CR increase could not be registered with our experiment. Since the CPM is constant and, accordingly, the CR is a linear function of *N*, the increase in intensity resulted from the increase in the *N* value (concentration of fluorescent molecules) from 0.24 to 6.0 in the entire experiment. Thus, the dynamics of the DAF-2 to DAF-2T transition induced by SNP were much faster than in the case of GSNO, although the concentrations of the dye and the NO donor were lower. The reaction dynamics depend on the kinetics of NO release from the donor and the binding to the dye; they are slower at low reagent concentrations.

The value of *N* measured in this FCS experiment (48 nM) was almost exactly equal to the actual value (50 nM). This range of nanomolar and potentially sub-nanomolar concentrations is not accessible in other methods and might correspond to the lower limit that is interesting in biology and medicine. For low concentrations of the NO donor in the nano- to micro-molar range, the kinetics of nitric oxide binding to the DAF-2 is obviously much slower than at the much higher (non-physiological) millimolar concentrations used in studies based on other methods. Thus, it is difficult to compare our results with some of the literature data. 

### 2.3. Measurements of Nitric Oxide Concentration in DAF-2 + Sodium Nitroprusside (SNP) and Glutathione (GSH) Solution in Other Solvents

#### 2.3.1. Measurements in the DAF-2 + SNP + GSH Solution in Water

Measurements in the DAF-2 (50 nM) + SNP (25 μM) + GSH (25 μM) solution, in water containing 100 mM NaCl, 20 mM Tris, and pH8, showed marginal changes in the parameters in comparison to the background. In a short time, the dye fluorescence intensity (CR) increased to 6.7 kcts/s, while *N* decreased to 0.27.

#### 2.3.2. Measurements in DAF-2 + SNP + GSH in a Buffered Physiological Salt Solution

At SNP (2.5 μM) and GSH (5 μM) concentrations, no increase in the fluorescent intensity was observed. An increase in the SNP concentration to 25 μM and GSH to 50 μM caused a fast increase in intensity to CR = 25.2 kcts/s and *N* to 3.3. At equal concentrations of SNP and GSH of 14 μM, there was a fast increase in CR to 14 kcts/s and *N* to 1.44. An increase in SNP concentration to 140 μM at a GSH concentration of 14 μM resulted in a fast increase in CR to 23.4 kcts/s and *N* to 4. Further increases of both SNP and GSH concentrations to 140 μM resulted in a fast increase in CR to 43.6 kcts/s and *N* to 8.5. After 3 h, the parameters remained unchanged.

Summarizing, in the DAF-2 + SNP + GSH solution in PBS and in the physiological salt solutions, fast increases in the dye’s fluorescence intensity (CR) from approximately 1 to over 40 kcts/s and in the number of fluorescent molecules up to 8 was observed. These strong increases occurred at DAF-2 concentrations of 50 nM and SNP and GSH concentrations of up to 140 μM. Since *N* = 1 corresponds to a fluorescent molecule concentration of 8 nM, the transition of all dye molecules to the fluorescent form corresponds to *N* ≈ 6.2. Thus, FCS indicated that practically all the DAF-2 molecules were converted to the fluorescent form.

In FCS experiments, the reaction with the DAF-2 is much less efficient. The kinetics of this process is much slower than reported in the literature for other methods at higher reagent concentrations in the μM-mM range. 

Since the physiological NO concentrations remained approximately at the nM-μM level, the question arises as to what extent the results of NO release obtained by other methods, at much higher concentrations of reagents, remain meaningful for understanding the processes in living cells. 

FCS offers new possibilities for investigation of NO production processes in cells by measuring very low concentrations of fluorescent products, down to below nM, and by providing local measurements in different cell sections due to the use of a confocal microscope.

To obtain reliable and reproducible results, the very slow kinetics of the reactions at very low concentrations must be taken into account.

### 2.4. Imaging of Fixed Cells

To investigate morphology and cytoskeletal organization during experiments, which play fundamental roles in the cells, stained F-actin fibers and nucleus images were examined after 24 h of culture in standard NUNC LabTek slides. 

**Figure 3:**
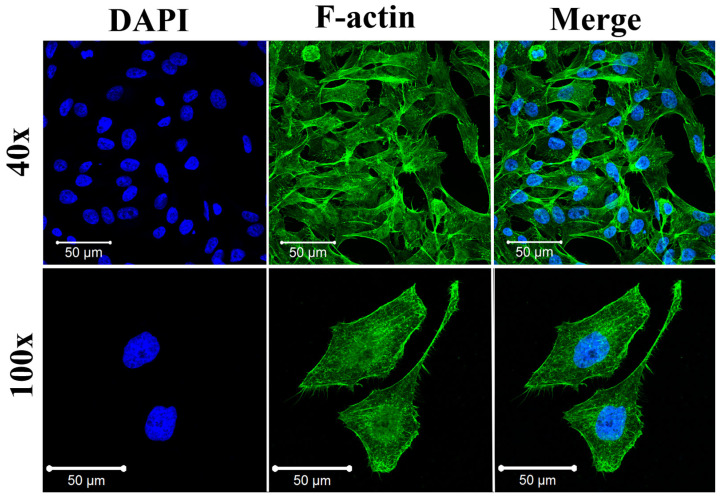
Immunofluorescent images of HUVEC cells cultured for 24 h on Lab-Tek. F-actin was stained by Alexa Fluor 488 Phalloidin (green) and cell nuclei were stained by DAPI (blue), respectively. Confocal laser scanning microscopes (CLSM) with a water immersion objective Zeiss 40×/NA 1.2 were applied.

**Figure 4:**
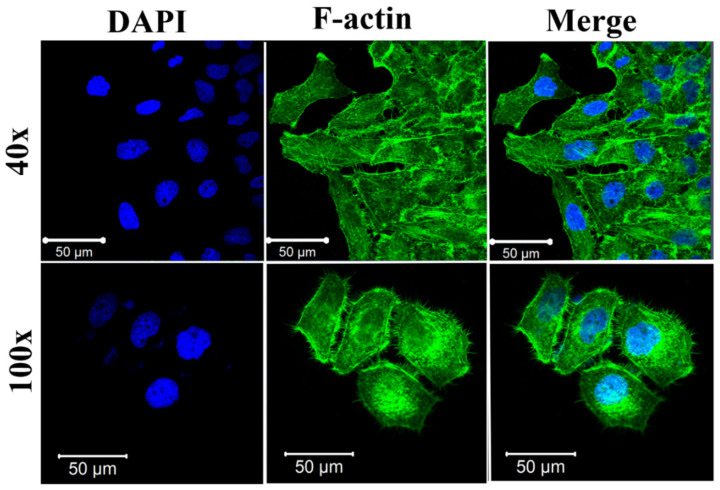
Immunofluorescent images of HeLa cells cultured for 24 h on Lab-Tek. F-actin was stained by Alexa Fluor 488 Phalloidin (green) and cell nuclei were stained by DAPI (blue), respectively. Confocal laser scanning microscopes (CLSM) with a water immersion objective Zeiss 40×/NA 1.2 were applied.

Both primary HUVEC cells and HeLa cells exhibited their proper morphologies, similar to those recently reported. [43] As shown in Figure 3, HUVEC cells exhibited proper endothelial cell phenotype characteristics, including the cytoskeletal structure and an elongated cell morphology. HeLa cells also had typical morphology, with an almost circular shape. Moreover, the cells were clearly attached to the glass slide’s surface, indicating their proper behavior (Figure 4).

### 2.5. Living Cells Imaging

In the next step, we investigated the effect of Calcein-AM on living HUVEC cells and HeLa cells cultured on Lab-Tek for 24 h according to the standard protocol (Calcein-AM (2 µM)).

**Figure 5:**
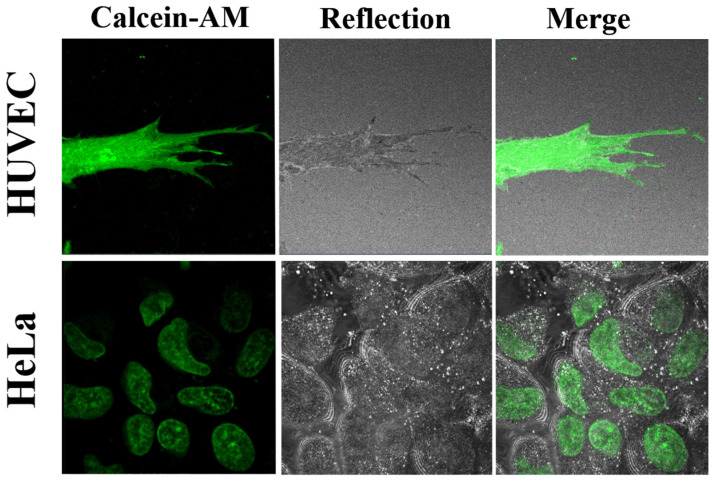
Representative CLSM images of HUVEC cells and HeLa cells stained by Calcein-AM using a standard protocol (Calcein-AM (2 µM) in fluorescent and reflected mode and merged. Confocal laser scanning microscopes (CLSM) with a water immersion objective Zeiss 40×/NA 1.2 were applied.

Cells cultured for 2 days on standard Lab-Tek glass surfaces were stained by Calcein-AM and imaged under 37 °C. As expected, Calcein-AM penetrated both living HeLa and HUVEC cells, generating uniform strong green fluorescent signals, indicating that the cells were viable (Figure 5). Based on Calcein-AM-stained living cells, HUVEC cells exhibited the typical elongated morphology with many filopodia extensions, indicating adhesion to the substrate and proper growth. HeLa cells showed characteristically rounded or irregular morphology. These results proved that both HeLa and HUVEC cells were viable, and that their passages were proper for further investigation by the DAF2-DA reagent.

#### 2.5.1. Measurements of NO Concentration in Living Cells

Finally, to investigate the detection of NO, we performed DAF2-DA imaging on living cells according to the standard protocol (10 µM of DAF2-DA). The DAF2-DA penetrates the cell membrane, where it becomes hydrolyzed by cytosolic esterases and traps NO into a product with a green fluorescence signal in the proximity of oxygen. For the method itself, the measurements of labeled dyes in living cells by FCS technique was previously described in the step-by-step protocol by Altan-Bonnet et al. [44].

**Figure 6:**
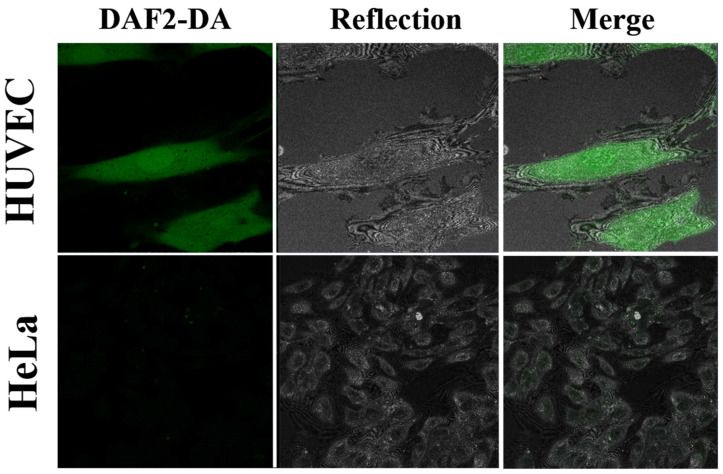
Representative CLSM images of HUVEC and HeLa cells of intracellular NO detection by the DAF2-DA using a standard protocol (10 µM) in fluorescent, reflected mode, and merged. Confocal laser scanning microscopes (CLSM) with a water immersion objective Zeiss 40×/NA 1.2 were applied.

The results showed that the fluorescence depended strongly on the cell type. DAF2-DA imaging showed that HUVEC cells exhibit strong green fluorescence from NO molecules. Additionally, the fluorescent signal in HUVEC was present in each cell consisting of homogenous distribution, which resembled the cytoskeleton. Some cells also had positively stained cell nuclei, probably due to NO diffusion, which was reported previously [45]. In contrast, we observed in HeLa cells almost no NO-induced fluorescence. A sparse, weak punctuated fluorescence pattern was observed, localized in the HeLa cells’ cytoplasm. (Figure 6).

**Figure 7:**
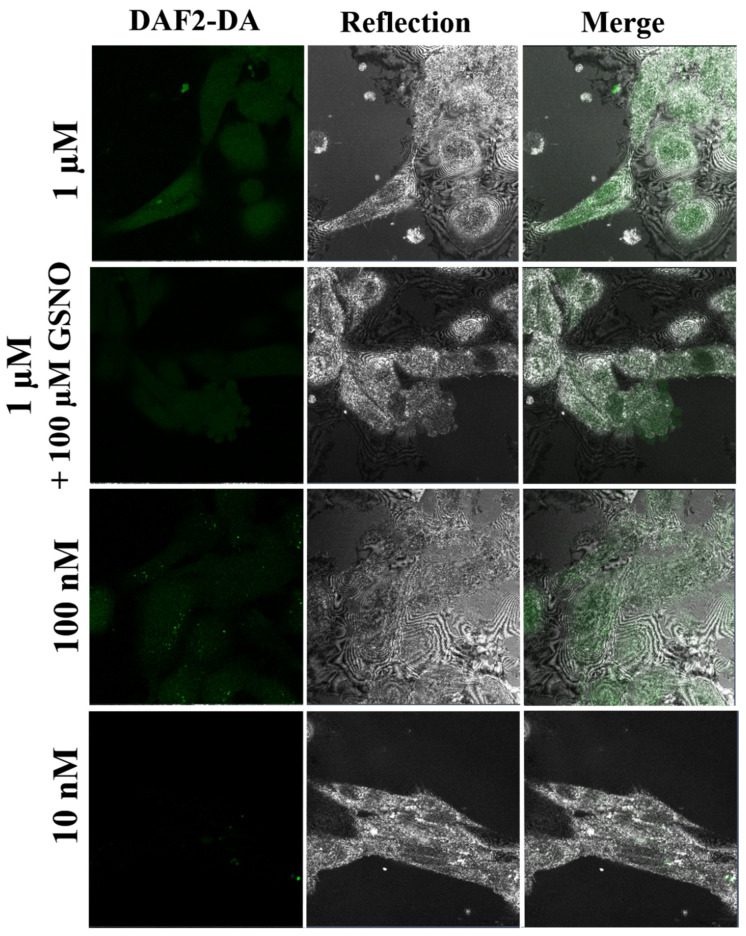
Representative CLSM images of intracellular NO detection by the DAF2-DA in HUVEC cells using much lower concentrations (1 µM, 100 nM, and 10 nM) of the DAF-2DA than the standard protocol (10 µM) in fluorescent (left panel) reflected mode (middle panel) and merged (bottom panel). Confocal laser scanning microscopes (CLSM) with a water immersion objective Zeiss 40×/NA 1.2 were applied.

**Figure 8:**
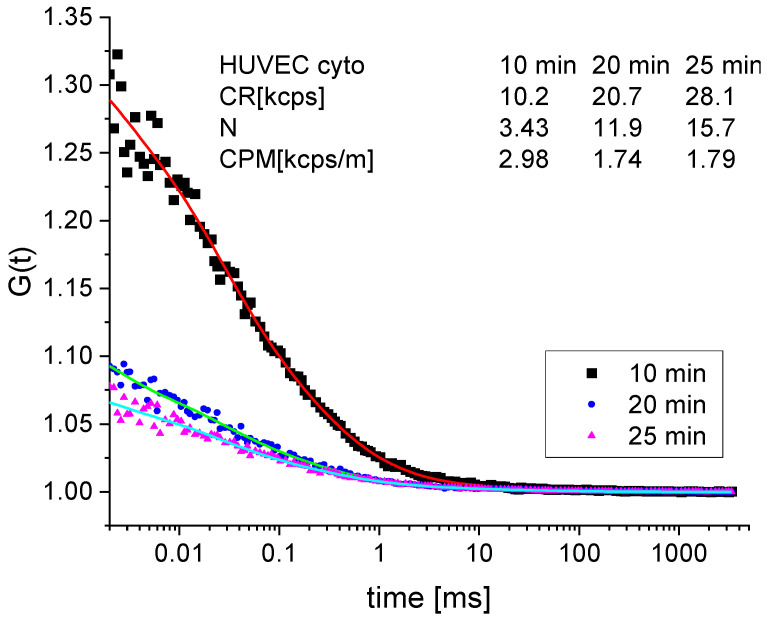
Correlation functions measured in the cytoplasm of an HUVEC cell at 10, 20, and 25 min, after adding the DAF-2DA dye (100 nM) to the cell-surrounding medium.

In the next step, we decreased the DAF-DA to 1 μM, 100 nM, and 10 nM, respectively. HUVEC cells treated by 1 μM DAF2-DA had weak green fluorescent signals but were still detectable by a confocal microscope, as presented in Figure 7. The overall green signal distribution was similar to that observed with the DAF-2DA under higher concentrations (according to the protocol, Figure 6) but localized primarily on the cytoplasm. To check whether the NO donor (GSNO) affected the HUVEC cells’ overproduction of NO, we treated the cells with the same detectable 1 μM of the DAF-2DA and 100 μM of GSNO. The resulting green signal was similar to that observed with 1μM DAF-2DA alone. Then, we substantially decreased the amount of the DAF2-DA to 100 nM, and the resulting signal from NO was much weaker than in the case of 1 μM of the DAF2-DA. Moreover, the green signal was distributed in a punctuated pattern, localized in the cytoplasm with bright spots at the cell corners. Finally, we used the lowest concentration of 1 nM of the DAF2-DA for visualization of NO produced by HUVECs. A small number of spot patterns were detected in the cytoplasm. All cells captured by the reflected mode had a proper elongated shape, suggesting proper adhesion and behavior. As shown in Fig 8, HUVEC cells incubated with DAF-2DA concentrations in the nanomolar range were almost invisible in the images obtained in the fluorescence mode. Thus, in the FCS experiments, images of unstained cells obtained in the reflection mode were used for proper localization of the confocal volume (Figure 7).

#### 2.5.2. FCS Measurements in HUVEC Cells

The optimization of the confocal volume in HUVEC cells’ cytoplasm was performed using Calcein-AM. The optimum position of the objective correction collar and all the instrument settings were maintained in the following DAF-2 measurements. Due to the optical heterogeneity of the cells, the confocal volume became more elongated, such that we estimated its size to be twice that of the size in the PBS environment. As a result, the reference concentration *c_ref_* corresponding to *N* = 1 was reduced to *c_ref_* ≈ 4 nM.

FCS measurements in HUVEC cells were performed at the DAF-2DA concentration in the cell-surrounding medium, amounting to 10 and 100 nM and 1 μM. The background fluorescence intensity of the HUVEC cytoplasm before adding the dye amounted to approximately 2.5 kcts/s, with no detectable correlation function. After adding the DAF-2DA at the highest concentration, the fluorescence intensity in the intracellular medium amounted to 0.8 kcts/s.

For a 1 μM concentration of the DAF-2DA in the cell-surrounding medium, the fluorescence intensity and the number of fluorescent molecules *N* in the HUVEC cells increased from the background value (2.5 kcts/s) to 105 kcts/s (*N* = 91, corresponding to 364 nM) after 60 s after the addition of the dye to the medium, and to the maximum value of 415 kcts/s (*N* = 240, corresponding to 0.96 μM) after 470 s.

For a 100 nM DAF-2DA concentration, the fluorescence intensity in the HUVEC cells increased from the background value to 10 kcts/s (*N* = 3.4/*c_m_* = 13 nM) in 10 min and to 47 kcts/s (*N* = 25/*c_m_* = 100 nM) in 35 min. The fast initial increase in the number/concentration of the fluorescent dye molecules in the HUVEC cells and a corresponding decrease in the contrast of the correlation function [*G*(0) – 1], where (*N* = 1/[*G*(0) – 1], can be seen in Figure 8, where during the time from 10 to 20 min after adding the dye, the contrast/number of molecules decreased/increased from 0.29/3.43 to 0.08/11.9, respectively.

For the lowest concentration (10 nM) of the DAF-2DA in the medium, the fluorescence intensity and the number of fluorescing dye molecules in the HUVEC cells increased from the background to 15 kcts/s (*N* = 5 /*c_m_* = 20 nM) after 6 min, and to 25 kcts/s (*N* = 12 / *c_m_* = 48 nM) after 20 min. Although counterintuitive, the concentration of the DAF-2T (a fluorescent form of the DAF-2), higher than the extracellular concentration of the DAF-2DA, was possible due to the membrane’s impermeability to the DAF-2. The DAF-2T were trapped within the cells. Another source of overestimation of *N* was the static fluorescence background arising from dye molecules that were trapped by immobile cell structures. 

Thus, one can see that the three-stage process—internalization of the non-fluorescent cell membrane permeable form of the DAF-2DA in the cell cytoplasm, a transformation of this dye form into another non-fluorescent form of the DAF-2 in the cytoplasm by esterases, and the reaction with nitric oxide to form a fluorescent DAF-2T form—is efficient and relatively fast. For DAF-2DA concentrations in the cell-surrounding medium from 10 nM to 1 μM, the process did not take longer than tens of minutes. Thus, we concluded that nanomolar nitric oxide concentrations can be measured in living cells using FCS.

#### 2.5.3. FCS Measurements in HeLa Cells

**Figure 9:**
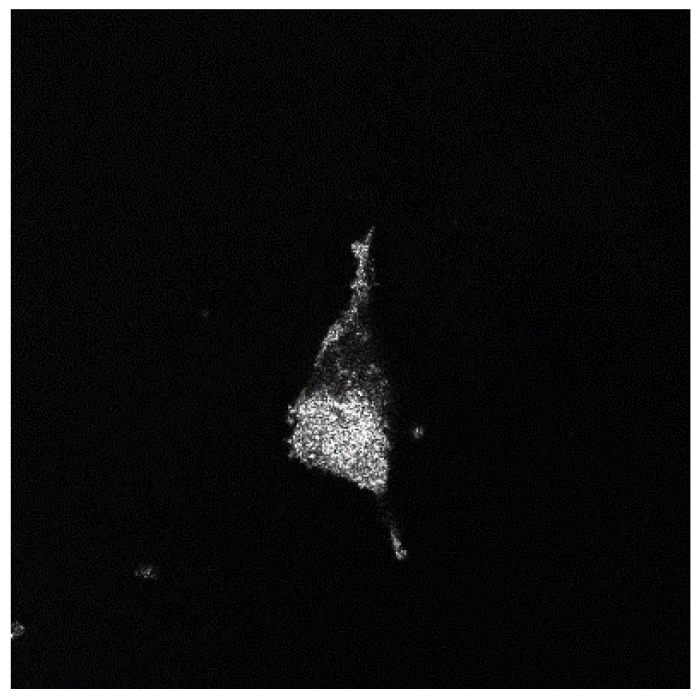
Confocal image of an HeLa cell in the reflection mode.

The HeLa cells were rinsed twice with pre-warmed PBS to reduce the background fluorescence. The background fluorescence intensity in the cells amounted to 3.2 kHz. The confocal imaging of the cells was done in the reflection mode, as presented in Figure 9.

First, the DAF-2DA was added to the cell-surrounding medium at a concentration of 1 μM and the fluorescence intensity in the cytoplasm was increased to 5.8 kHz.

**Figure 10:**
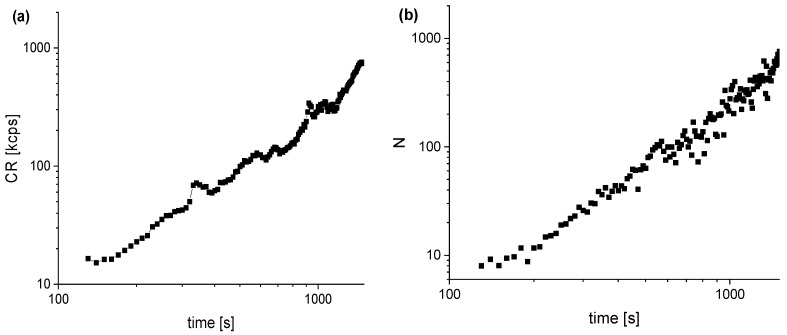
Time dependence of (**a**) the fluorescence intensity and (**b**) the number of fluorescing dye molecules measured in the HeLa cytoplasm after adding GSNO to the DAF-2 containing HeLa cells.

After subsequently adding 100 μM GSNO to the cell-surrounding medium, the imaging scan was performed, and then a series of correlation functions were measured every 10 s. The time dependence of the fluorescence intensity and the number of fluorescent dye molecules in the cytoplasm increased, as shown in Figure 10a,b. The correlation functions were analyzed using a three-component model plus triplet. The component corresponding to free dye had a diffusion time of 22–24 μs.

GSNO was added approximately 30 min after adding the DAF-2DA. Based on our measurements in the HUVEC cells, we can assume that the dye concentration in the cytoplasm reached the saturation concentration before GSNO was added. Thus, the measured kinetics (Figure 10) reflects the dynamics of three processes: internalization of GSNO in the cytoplasm, the release of nitric oxide, and the reaction of nitric oxide with the DAF-2 (present in the cells) to form the fluorescent form of the DAF-2T.

**Figure 11:**
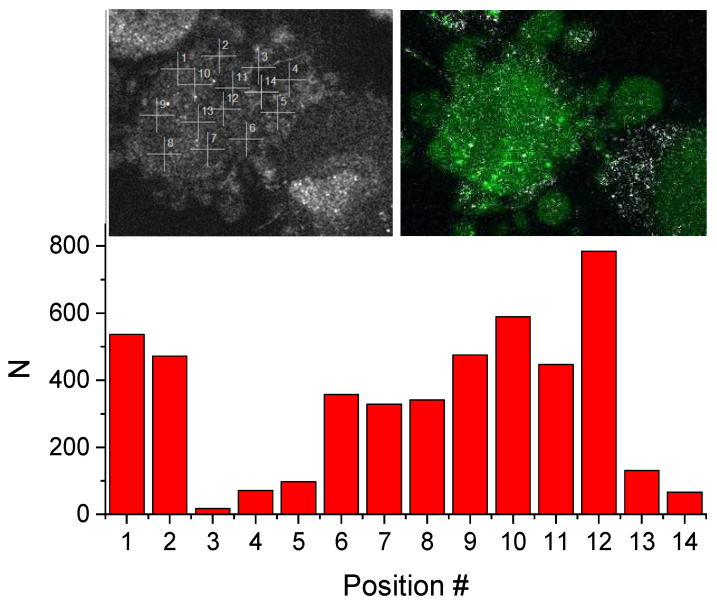
Parameter *N* values measured in different positions in the HeLa cell. Inset left shows the positions; inset right shows the confocal scan of the cell in the fluorescence mode.

To check the distribution of nitric oxide in a living cell, the correlation functions were measured in different positions in the cell, and the values of parameter *N*, being a measure of dye concentration, are shown in Figure 11. The positions and the confocal scan of the cell in the fluorescence mode are shown in the inset.

One can see a substantial fluctuation of the fluorescence intensity in different cell regions, which, in part, might be caused by substantial optical heterogeneities due to the presence of the cell cytoskeleton and organelles, resulting in a distortion of the confocal volume. In positions 3, 4, 5, and 14, the confocal volume was probably located outside the cell, which is supported by the fact that the correlation functions could be fitted with a single component model at those positions.

## 3. Materials and Methods

### 3.1. Materials

Glutathione (GSH), hydrochloric acid, sodium nitrite, sodium nitroprusside (SNP), 5-diaminofluorescein (DAF-2), 4,5-diaminofluorescein diacetate (DAF-2 DA), as well as all the solvents, were purchased from commercial suppliers and used without further purification (Sigma-Aldrich, St. Louis, MO, USA, Avantor Performance Materials Poland S.A, Gliwice, Poland). Water purified with Mili-Q^®^ system (Merck Millipore, Merck KGaA, Darmstadt, Germany) was used for all applications (resistivity 18 MW cm^−1^).

#### S-nitrosoglutathione Preparation

S-nitrosoglutathione (GSNO) was synthesized according to the method that is well-described in the literature [46]. To a stirred and ice-cold solution of glutathione in water containing HCl, an appropriate amount of sodium nitrite was added in one portion. After 40 min, the reaction mixture (which turned red) was treated with acetone and stirred for a further 10 min. A pale red precipitate was filtered and washed three times with ice-cold water, acetone, and ether. The structure of the synthesized GSNO was confirmed by nuclear magnetic resonance and infrared spectroscopy.

### 3.2. Cell Culture

Two human cell lines were chosen to investigate NO detection: primary human umbilical vein endothelial cells (HUVEC) (ATCC, Manassas, VA, USA), and cervical cancer HeLa cells (ATCC, Manassas, VA, USA). The primary HUVEC cells were cultured in endothelial cell growth medium (ATCC, Manassas, VA, USA), containing 2% FBS and supplemented with an endothelial cell growth supplement mix containing: 50 µg/mL ascorbic acid, 1 µg/mL hydrocortisone, 0.75 units/mL heparin sulfate, 10 mM L-glutamine, 15 ng/mL rh IGF-1, 5 ng/mL rh FGF basic, 5 ng/mL rh EGF, and 5 ng/mL rh VEGF. HeLa cells were cultured in Dulbecco’s modified Eagle’s medium, DMEM (Sigma-Aldrich, St. Louis, MO, USA), supplemented with antibiotics (penicillin 100 U/mL, streptomycin 100 µg/mL) containing 10% fetal bovine serum (FBS) (Sigma-Aldrich, St. Louis, MO, USA). HUVEC cells and HeLa cells were cultured as a monolayer on sterile cell culture flasks and maintained in a humidified atmosphere containing 5% CO_2_ at 37 °C. HUVEC and HeLa cells at passages 2–4 were used in the experiments. The medium was changed every 2 days. When the cell culture reached the required confluence (70%), the cells were washed with Balanced Salt Solution (HBSS, Sigma-Aldrich, St. Louis, MO, USA), trypsinized with trypsin-EDTA (Sigma-Aldrich, St. Louis, MO, USA), and counted using an automated cell counter (BioRad, Hercules, CA, USA).

### 3.3. Detection of Nitric Oxide Using DAF-2 and DAF-2DA

**Figure 12:**
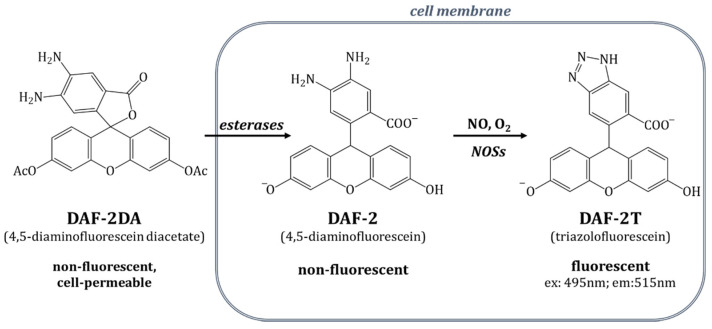
Schematic representation of diaminofluorescein-based dye behavior in a living cell.

Diaminofluorescein diacetate (DAF-2DA) is a membrane-permeable, non-fluorescent organic compound, allowing free nitric oxide measurement inside a cell. After permeating the membrane, the diacetate groups of DAF-2DA were hydrolyzed by cytoplasmic esterases to form diaminofluorescein (DAF-2), which is also non-fluorescent. In the presence of nitric oxide released by NOSs (or external stimuli in the case of NO donors), this form transformed to a triazole derivative of fluorescein (DAF-2T), which was observed at an excitation length of 488 (495) nm and an emission of 515 nm. Importantly, both forms, the DAF-2 and the DAF-2T, were retained inside the cell. The scheme of the DAF-2-based dyes acting inside living cells is presented in Figure 12. The minimal concentration of nitric oxide that can be noticed in the case of the DAF-2 is 5nM [24].

### 3.4. Fluorescence Correlation Spectroscopy (FCS)

Fluorescence correlation spectroscopy (FCS) measures the amplitude and rate of fluorescence fluctuations in a sub-micron volume defined by a confocal microscope. In the most common case, when the source of fluorescence fluctuations is the Brownian motion of fluorophores, FCS allows for estimation of their translational diffusion coefficient *D* and their concentration, measured as the number of particles *N* in the so-called confocal volume seen by the photodetector. The signal-to-noise ratio in FCS is optimal when only one particle is present in the confocal volume. Therefore, objectives with large numerical apertures are used in FCS setups to provide optimum signal collection efficiency. The optimum fluorophore concentration typically is in the range of single nanomoles/dm^3^. As both the mean fluorescence signal count rate (CR) and the fluorophore concentration are measured, FCS provides a unique possibility to estimate the molecular brightness of a single fluorophore. Moreover, its characterization is complemented by the information on its diffusion coefficient, obtained in the same experiment. Technically, a time correlation function *G(t)* of the fluorescence intensity is calculated online by a hardware correlator, and the parameters of an appropriate model are fitted to that function. The model should include all physical and chemical processes that influence the fluorescence intensity in the studied system.

The in vitro FCS experiments were performed using the ConfoCor2 system (Zeiss, Jena, Germany). The excitation wavelength was set to 488 nm, and a band-pass BP 505-550 filter was used to select the fluorescence band. A water immersion objective Zeiss 40×/NA 1.2 with a correction collar was applied to focus the incident laser beam and to collect the fluorescence signal. In the analysis of the correlation functions (CFs), their first points were removed (up to a delay time of 1 μs) due to the influence of the after-pulse effect. The laser power was set to 0.5 % at the lowest available current (25 % maximum), preventing bleaching and reducing the triplet fraction contribution.

Correlation functions were analyzed using the standard model, including diffusion and triplet contributions, as follows:

(1) $$G\left(t\right)=1+\frac{1}{N}\left(1+\frac{1}{1-T}e^{-\frac{t}{\tau_{T}}}\right){\left(1+\frac{t}{\tau_{D}}\right)}^{-1}{\left(1+\frac{t}{SP^{2}\tau_{D}}\right)}^{-0.5},$$

where *N* is the mean number of fluorophores in the confocal volume, *T* is the triplet amplitude, *τ_T_* is the triplet relaxation time, *τ_D_* is the diffusion characteristic time, *SP* = *σ_1_/σ_2_* is the confocal volume aspect ratio, and *σ_1_*, *σ_2_* are the dimensions of the confocal volume in directions parallel and perpendicular to the beam axis, respectively. The mean fluorescence intensity measured by the photon count rate (CR) was recorded during each measurement. The ratio CR/*N* was calculated to estimate the molecular brightness of the fluorophores, measured by counts per molecule (CPM).

To convert parameter *N* into the absolute concentration of fluorophores in solution, we decided to calculate *σ*_2_ by measuring the diffusion time *τ_D_* of a standard dye whose diffusion coefficient *D* is known to be *σ_2_^2^* = *4Dτ_D_*. The confocal volume *V* can be calculated as follows:

(2) $$V=\pi^{\frac{3}{2}}\sigma_{1}\sigma_{2}^{2}=\pi^{\frac{3}{2}}\sigma_{2}^{3}SP,$$

The absolute molar concentration *c_m_* is

(3) $$C_{m}=\frac{N}{N_{A}V},$$

where *N_A_* is the Avogadro number and *V* must be given in dm^3^.

#### 3.4.1. FCS Measurements and Confocal Imaging

FCS experiments in cells were performed using a confocal laser scanning microscope (Zeiss, LSM 780, Jena, Germany) equipped with the ConfoCor3 unit. The excitation was performed with a 488 nm Argon ion laser and a long pass emission filter LP 505 nm. A water immersion objective Zeiss 40x/NA 1.2W selected for FCS was used for imaging and FCS measurements. To avoid background signals, no additional cell staining was used in all FCS experiments. As our microscope did not have a transmission detector, the shape of cells was recorded using the reflection mode, and the actual measurement spots were chosen based on such images. The laser power was adjusted to the highest level that allowed for bleaching to be avoided. To extend the cell’s lifetime, we used a simple homemade heating stage, allowing us to maintain ~37 °C in the Nunc™ Lab-Tek™ chambered coverglass. Only up to two wells in every LabTek plate were used, to avoid long exposure of cells to the environment outside the culture incubator. The FCS measurements were performed in 10 s “modules,” which could be integrated in longer periods in the case of slower reaction kinetics.

#### 3.4.2. FCS Measurements and Imaging in Cells

Two human cell line types were chosen: primary human umbilical vein endothelial cells (HUVEC) and an immortal human epithelial cell line, cervical cancer HeLa cells. Among the most studied endothelial cells that release NO are HUVECs [10,47,48]. To verify that the DAF-2DA can also quantify NO produced by a mixed population of primary endothelial cells, we employed HUVEC cells pooled from multiple donors. As a negative control, we used HeLa cells known for having no eNOS expression [49]. Experiments were conducted with primary HUVEC cells and HeLa cells from passages 2 to 4. Independently, HUVEC cells and HeLa cells were seeded in LabTek-8 well chamber slides at a density of 3 × 10^4^ cells/well in a proper growth medium and incubated overnight at 37 °C in the humidified conditions described above.

##### F-actin/DAPI Imaging of Cell Morphology Visualization

To evaluate the cell morphology by F-actin detection, after 24 h of culture, cell media were removed, and the cells were washed twice in phosphate-buffered saline (PBS, Sigma Aldrich, St. Louis, MO, USA) and fixed in 3.7% paraformaldehyde in PBS solution for 15 min. Then, all the samples were permeabilized by 0.1% Triton X-100 in PBS. To visualize the F-actin cytoskeleton, Oregon Green 488 Palloidin (Thermo Fisher Scientific, Waltham, MA, USA) solution in PBS containing 1% BSA was added to the samples and incubated for 20 min at room temperature (RT = 21 °C). Finally, the solution was removed, and the samples were again washed twice in PBS. For cell nuclei visualization, the samples were incubated with DAPI (4,6-diamidyno-2-fenyloindol) (Thermo Fisher Scientific, Waltham, MA, USA) solution for 5 min at room temperature. The stained cells were imaged under a confocal laser scanning microscope (Zeiss, LSM 780) with an excitation laser wavelength measured at λ= 488 nm and peak emission measured at λ = 520 nm, and λ = 360 nm and λ = 460 nm for actin and nuclei, respectively. A 40×/NA 1.2 water immersion objective was used. All the experiments were repeated in triplicate (*n* = 3).

##### Calcein-AM Imaging of Living Cells

For live cell imaging experiments, after 24 h of incubation, the cells were labeled with Calcein-AM for the staining of live cells. A solution of 2 μM of calcein-AM (as a control for the standard staining procedure), in PBS was incubated with cells for 30 min at 37 °C. We used much lower concentrations of Calcein-AM solutions of 2 and 20 nM for FCS measurements. After that, cells were washed by PBS, and the medium was replaced with a new, pre-warmed medium taken from the incubator. Next, the cells were immediately visualized on a CLSM with a 40×/NA 1.2 water immersion objective and excitation laser wavelengths at λ = 488 nm for the Calcein-AM and an emission range of λ = 500−580 nm.

##### DAF-2DA Imaging of Nitric Oxide

For live cell imaging experiments, after the incubation, the cells were washed and incubated with 5 μM of the DAF-2DA (Sigma, St. Louis, MO, USA) at 37 °C for 30 min, as a control for the standard staining procedure. We used a lower concentration of the DAF2-DA solutions for FCS measurements, in the range of 10 nM to 1 μM. Then, the cells were washed repeatedly, and the medium was replaced with a new, pre-warmed medium taken from the incubator. Fluorescence images and FCS measurements were captured immediately on a confocal laser scanning microscope (CLSM, Zeiss LSM 780) with a 40×/NA 1.2 water immersion objective and excitation laser wavelengths at λ= 488 nm for the DAF2-DA and an emission range of λ = 500−580 nm.

## 4. Conclusions

Fluorescence correlation spectroscopy was used to study the efficiency and kinetics of the transition of the non-fluorescent form of the dye DAF-2 to its fluorescent form DAF-2T, induced by NO released from two NO donors, GSNO and SNP in PBS solutions, and released from GSNO in living HUVEC and HeLa cells. It was shown that the fluorescence of a single DAF-2T molecule is sufficient to determine the fluorophore concentration in the sub-nanomolar to micromolar range that is characteristic for NO concentrations in vitro.

In the case of GSNO, it was shown that efficient conversion of the dye to the DAF-2T form in PBS solutions requires DAF-2 concentrations in the sub-micromolar (250 nM) and NO donor concentration in the micromolar (70 μM) range. At these concentrations, the fluorescence intensity was proportional to the DAF-2T concentration and slowly increased exponentially by more than a factor of 10 with a rise time of 474.7 min. Even at these relatively high (for FCS) concentrations and the high excess of the NO donor (GSNO), only about half of the DAF-2 molecules were converted to the fluorescent DAF-2T form.

The concentration of the DAF-2T in PBS solutions increased much faster (exponential rise time 9.3 s) when SNP was used as the NO donor, although the DAF-2 (50 nM) and SNP (14 μM) concentrations were much lower than in the case of GSNO. Thus, we can see that the increase in the DAF-2T concentration (obtained via FCS) depends on the rate of NO release from NO donors and the concentration of reactants. In our experiment, the concentration of reactants was lower than the concentration that is usually used in other methods, which slowed down the reaction kinetics. An important question arises: how can the results of NO detection in experiments using other methods, usually with (much) higher reactants concentrations, be applied to lower concentrations similar to physiological conditions.

Our experiments in PBS solutions also indicated that at low concentrations, the time required to reach the final DAF-2T concentration might be long (i.e., an order of tens of hours) and this factor must be taken into account.

Our studies on living HUVEC cells exposed to the non-fluorescent cell membrane permeable DAF-2DA showed that the formation of a fluorescent form of the DAF-2T in the cytoplasm achieved by a three-step reaction—internalization of the DAF-2DA in the cell cytoplasm, transformation of this dye form into another non-fluorescent form of the DAF-2 by esterases, and reaction with nitric oxide to form a fluorescent DAF-2T—is efficient and rather fast, and for the DAF-2DA concentrations in the extracellular medium from 10 nM to 1 μM does not take longer than tens of minutes.

Further, in living HeLa cells exposed first to the DAF-2DA and subsequently, to the NO donor GSNO, it was possible to measure the kinetics of increase in nitric oxide concentration in the cell cytoplasm, which corresponds to the combined effect of three processes: internalization of GSNO in the cytoplasm, the release of nitric oxide, and the reaction of nitric oxide with the DAF-2 to form the fluorescent form of the DAF-2T detected in the FCS experiment.

Thus, we can conclude that nanomolar concentrations of nitric oxide, produced either in the cell or transferred into the cell from an extracellular medium, can be measured in living cells using FCS.

We have also shown that the detection of nitric oxide by means of the DAF-2DA or the DAF-2 is much more efficient and faster in living cells (where the DAF-2DA is used) than in PBS solutions (where the DAF-2 is applied). Since the conversion of the non-fluorescent forms of the DAF-2DA in cells and the DAF-2 in solutions, respectively, to the fluorescent form of the DAF-2T under the influence of NO is, in both cases, a multi-step reaction, which additionally depends on the efficiency and speed of NO release from the donor (GSNO), it is impossible at present to identify the speed/efficiency limiting steps of the reactions.

Since a confocal microscope was used in the FCS experiment, limiting the observation volume to the sub-micrometer range, the distribution of local NO concentration in a cell may also be studied.

## Data Availability

Data are available from the corresponding authors.

## Sample Availability

Samples of the compounds are available from the authors.
